# Supplementary material for: The interactome of intact mitochondria by cross-linking mass spectrometry provides evidence for coexisting respiratory supercomplexes
Source: Mol Cell Proteomics. 2017 Dec 8;17(2):216–32. doi: 10.1074/mcp.RA117.000470 (PMC5795388; doi:10.1074/mcp.RA117.000470)
Supplement: Supplemental Data [file supp_RA117.000470_133922_0_supp_23978_fzffwf.zip › spectra_annotation/mito_DR_spectra_annotation/256-1-4-1-9-1.pdf]

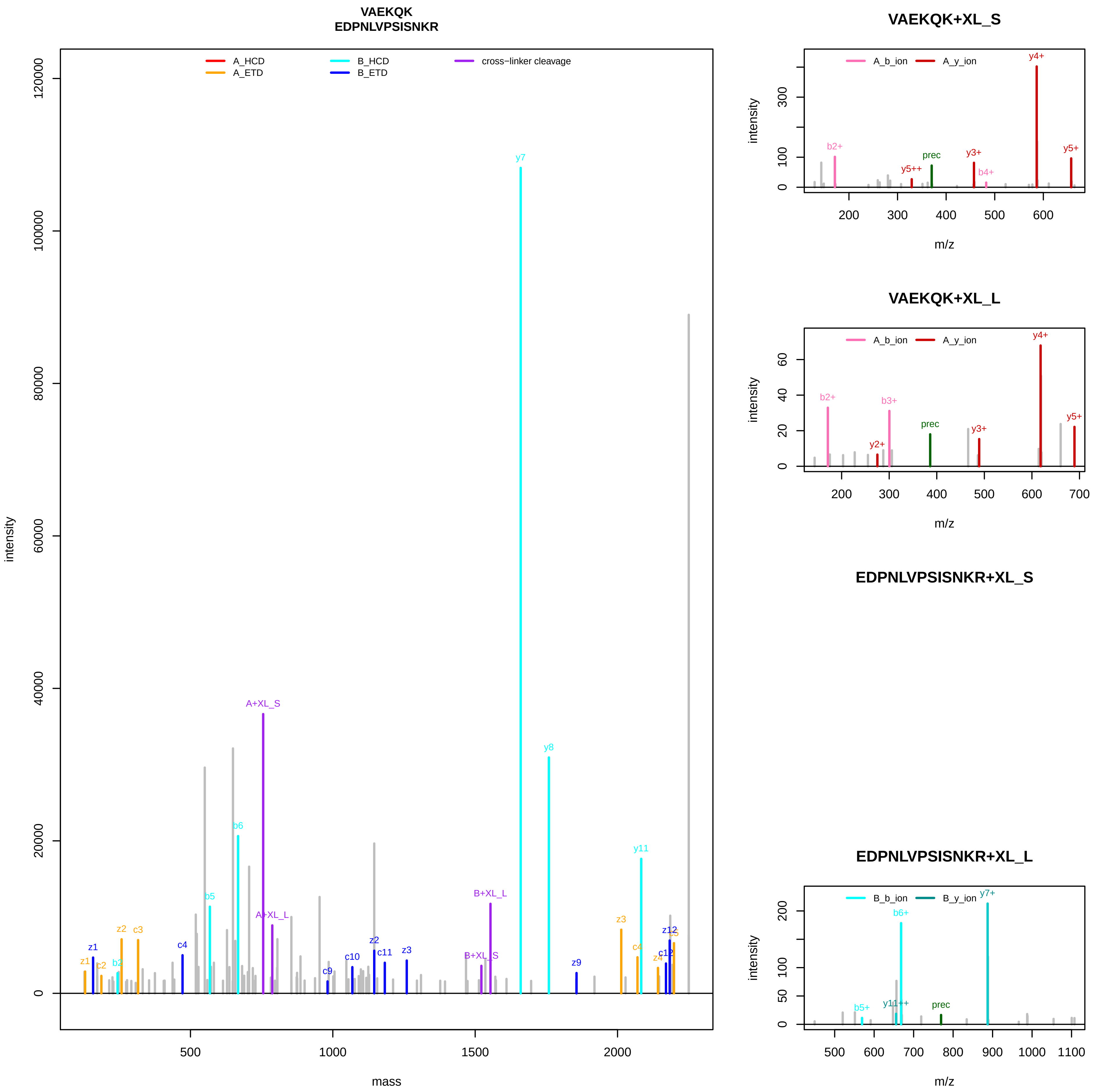
VAEKQK
EDPNLVPSISNKR
A_HCD
A_ETD
B_HCD
B_ETD
cross−linker cleavage
y7
y8
y11
b5
b6
b2
A+XL_S
A+XL_L
B+XL_L
B+XL_S
z1
z2
z3
z4
z9
z12
c2
c3
c4
c9
c10
c11
c12
intensity
mass
VAEKQK+XL_S
VAEKQK+XL_L
EDPNLVPSISNKR+XL_S
EDPNLVPSISNKR+XL_L
A_b_ion
A_y_ion
B_b_ion
B_y_ion
b2+
b3+
b4+
b5+
b6+
y2+
y3+
y4+
y5+
y5++
y7+
y11++
prec
m/z
